# Bioinformatics analysis and identification of potential genes related to pathogenesis of cervical intraepithelial neoplasia

**DOI:** 10.7150/jca.38211

**Published:** 2020-02-03

**Authors:** Xue Zhang, Jian Bai, Cheng Yuan, Long Long, Zhewen Zheng, Qingqing Wang, Fengxia Chen, Yunfeng Zhou

**Affiliations:** 1 Department of Radiation and Medical Oncology, Zhongnan Hospital, Wuhan University, Wuhan, Hubei 430071, P.R. China; 2 Department of Gastrointestinal Surgery and Department of Gastric and Colorectal Surgical Oncology, Zhongnan Hospital of Wuhan University, Wuhan, Hubei, China

**Keywords:** cervical intraepithelial neoplasia, bioinformatical analysis, microarray, differentially expressed genes

## Abstract

The aim of this study was to explore and identify the key genes and signal pathways contributing to cervical intraepithelial neoplasia (CIN). The gene expression profiles of GSE63514 were downloaded from Gene Expression Omnibus database. Differentially expressed genes (DEGs) were screened performing with packages in software R. After Gene ontology terms, Kyoto Encyclopedia of Genes and Genomes (KEGG) pathway enrichment analyzing, and Gene set enrichment analysis (GSEA), weighted gene co-expression network analysis (WGCNA) was used to analyze these genes. Then sub-modules were subsequently analyzed base CIN grade, and protein-protein interaction (PPI) network of DEGs were constructed. 537 DEGs were screened in total, consisting 331 up-regulated genes and 206 down-regulated genes in CIN samples compared to normal samples. The most DEGs were enriched in chromosomal region in cellular component (CC), organelle fission inbiological process (BP) and ATPase activity in molecular function (MF). KEGG pathway enrichment analyzing found the DEGs were mainly concentrated in 10 pathways. The results of GSEA mainly enriched in 4 functional sets: E2F-Targets, G2M-Checkpoint, Mitotic-Spindle and Spermatogenesis. A total of 6 modules were identified by WCGNA. Subsequently, grey module was the highest correlation (Cor=0.78, P=5e-22) and 31 genes were taken as candidate hub genes for CIN high grade risk (weighted correlation coefficients >0.80). Finally, diagnostic analysis showed that in addition to CCDC7, the expression levels of the remaining 13 DEGs have a high diagnostic value (AUC>0.8 and P<0.05). These findings provided a new sight into the understanding of molecular functions for CIN.

## Introduction

The formation of cervical cancer is a continuous process from inflammation to cervical intraepithelial neoplasia (CIN), and finally to invasive cancer, which takes 10 to 25 years^1^-^3^. CIN is regarded as a potentially premalignant transformation of squamous cells of the cervix. According to the composite data for the natural history of CIN, CIN1 is likely to regress in 60% of cases, persist in 30%, progress to CIN 3 in 10%, and progress to invasion in 1%^4^. Two high-risk HPV subtypes (types 16 and 18) themselves produce two proto-oncoproteins, E6 and E7, which are key to their disease^5^.

In recent years, many studies have focused on the diversity or heterogeneity of various solid tumor types^6^,^7^. Gene expression network patterns are more complex in cancer cells and tumors than in normal cells and organs^8^-^11^. In the study of Banerji et al.^8^, signaling entropy has been found to be significantly higher in cancer cells, especially cancer stem cells, than in normal cells, thereby helping to distinguish them.

In this study, genes from CIN and normol samples were analyzed and screened for differentially expression, from microarray datasets (GSE63514), using bioinformatics. Functions and signal pathway enrichments of differentially expressed genes (DEGs) were analyzed. Moreover, WGCNA explored the genes modules were associated with CIN grade. Finally, identifying the biological function of the hub genes and pathways, this study may offer a better insight of potential molecular mechanisms to explore novel therapeutic strategies for CIN.

## Methods

### Data Procession

The gene expression profiles of GSE63514 (https://www.ncbi.nlm.nih.gov/geo/query/acc.cgi?acc=GSE63514) submitted by den Boon J et al. was downloaded from the Gene Expression Omnibus (GEO) database. The GSE63514 was an expression profiling based on GPL570 platform (Affymetrix Human Genome U133 Plus 2.0 Array) and contained 128 samples (24 normal samples, 76 CIN samples and 28 cervical cancer samples). All samples were taken from flash-frozen biopsy and cryosectioned. This study mainly focused on the Screening for differentially expressed genes (DEGs) between CIN and normal samples, therefore, the 28 cervical cancer samples were not included.

**Figure 1:**
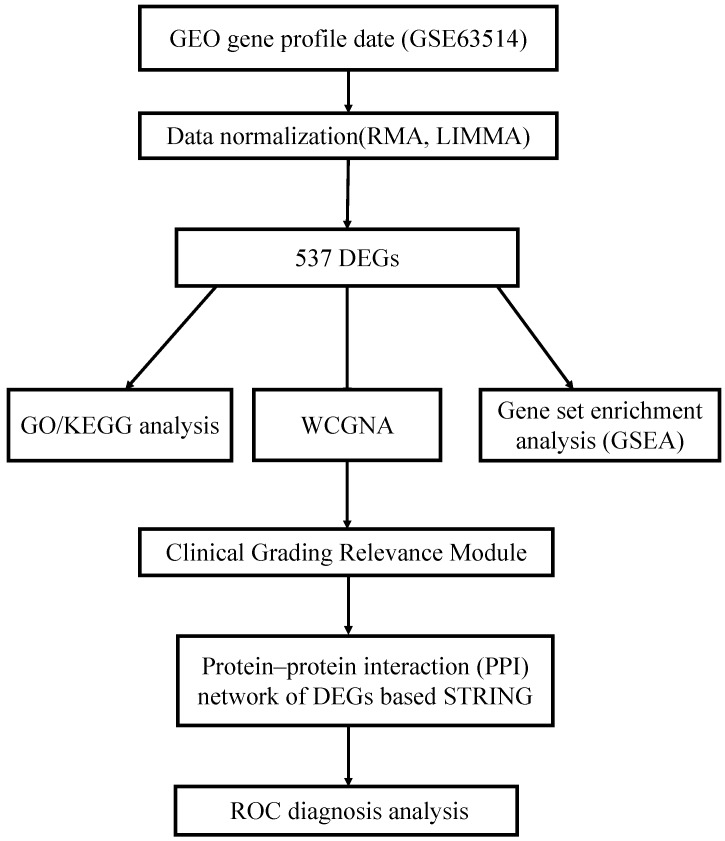
The flowchart of the integrated analysis and functional validation.

Prior to bioinformatics analysis, we first mapped the array probes to the respective Gene ID by using the array annotations. If a probe matches multiple genes, the probe will be deleted. If a gene matches multiple probes, we will calculate its average value. A proper threshold was settled based on the amount of genes filtered out. A workflow of this study was indicated in Fig. 1.

### Analysis of microarray datasets

Limma package^12^ in R/Bioconductor software was used to compare CIN sample with its normal sample. In addition, normalization and log_2_ conversion were carried out for each GEO dataset to filter out the final DEGs. The filtration conditions are as follows: |log2FC| ≥1 and adjust P‐value(AdjP-value) < 0.05.

### Enrichment analysis of gene function and pathways

The ClusterProfiler is an ontology-based R package, it applies the biological terms classification and enrichment analysis to the comparison of gene clusters to better understand the higher order functions of biological system^13^. DAVID^14^ (http://david.abcc.ncifcrf.gov/), a common functional annotation tool of bioinformatics resources was utilized to distinguish the biological attributes such as biological process (BP), cellular component (CC) and molecular function (MF) of important DEGs. Moreover, Kyoto Encyclopedia of Genes and Genomes (KEGG)^15^ (http://www.genome.jp/kegg/) pathway enrichment analysis was used to discern the crucial pathways significantly. Adj*P*-value <0.05 was set as the cut-off criterion for the significant enrichment.

### Gene set enrichment analysis (GSEA)

The enrichment analyses were conducted to detect whether a series of priori defined biological processes was enriched. The enriched pathways were arranged in the order of their normalized enrichment scores (NESs), and FDR < 0.05 was chosen as the cut-off criteria.

### Construction of gene co-expression network

Firstly, the quality of the DEGs of GSE63514 was checked through R package. Then, the scale-free gene co-expression network was constructed through the “WGCNA” package. Pearson's correlation matrices were calculated and a weighted adjacency matrix was constructed through a power function amn = |cmn|β (cmn means Pearson's correlation between gene m and gene n; amn = adjacency between gene m and gene n). Afterwards, the most appropriate soft - thresholding parameter (β) was chosen to transform the adjacency matrix into a topological overlap matrix (TOM), so that modules including similar genes were identified. Module eigengenes (MEs) was defined as the most principal component and clarify all genes into a single characteristic expression profile. The correlation between module exigencies (MEs) was defined as the dominating component of gene module and clinical traits to identify the correlative module. The module highly related to given clinical characteristics was selected for further analysis.

## Results

### Identification of DEGs

The clinical parameters are shown in Tab S1. A total of 100 tissues were divided into 76 CIN and 24 normal samples in GSE53757. After integrated analysis, 537 DEGs (|log2 FC| ≥ 1 and AdjP-value < 0.05) were screened in total, consisting 331 up-regulated genes and 206 down-regulated genes in CIN samples compared to normal samples. Volcano plots (Fig. S1) were visualized to show the correlation between DEGs.

### GO, pathway enrichment analysis and GSEA of DEGs

**Figure 2:**
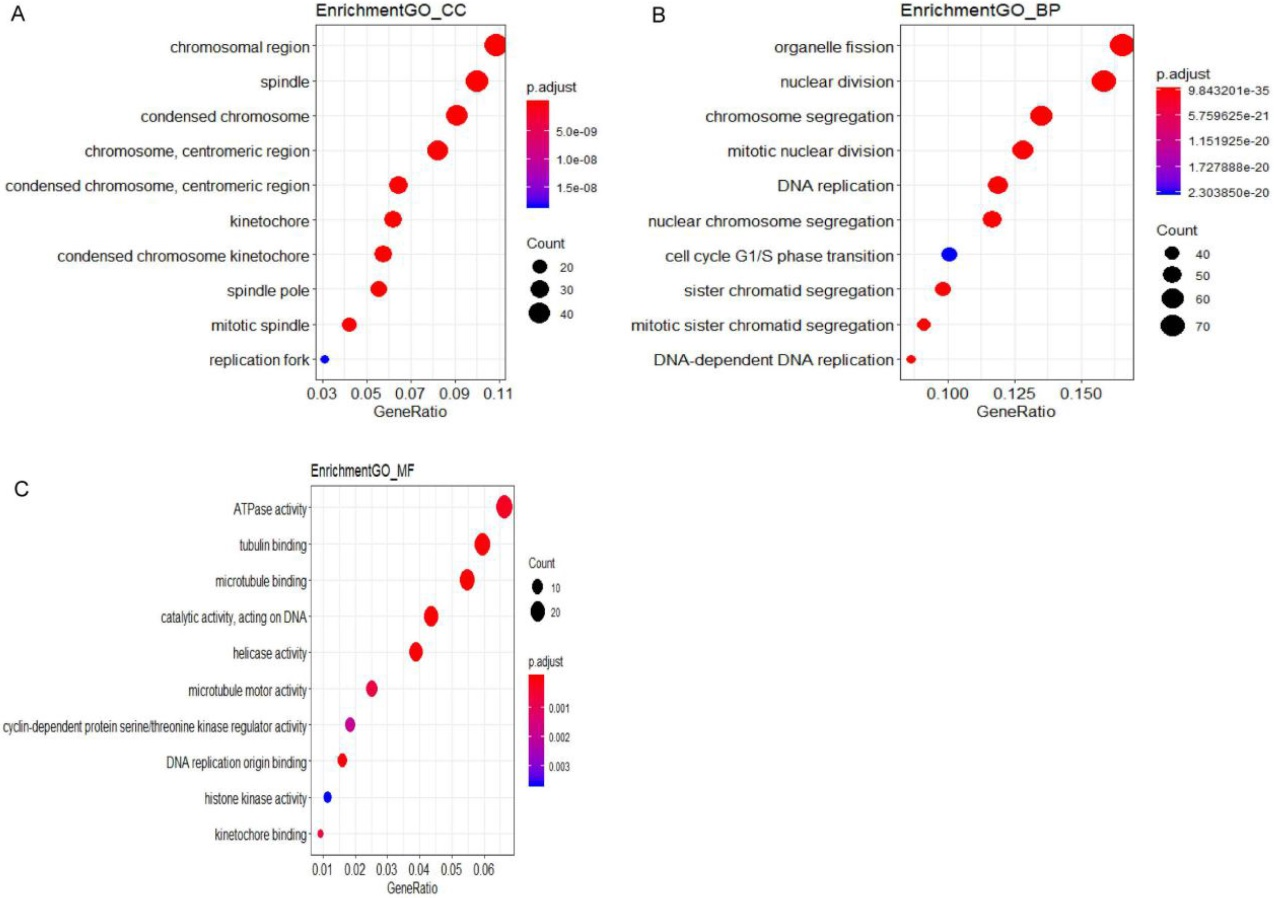
GO analysis and the significantly terms of differentially expressed genes (DEGs) in CIN.

**Figure 3:**
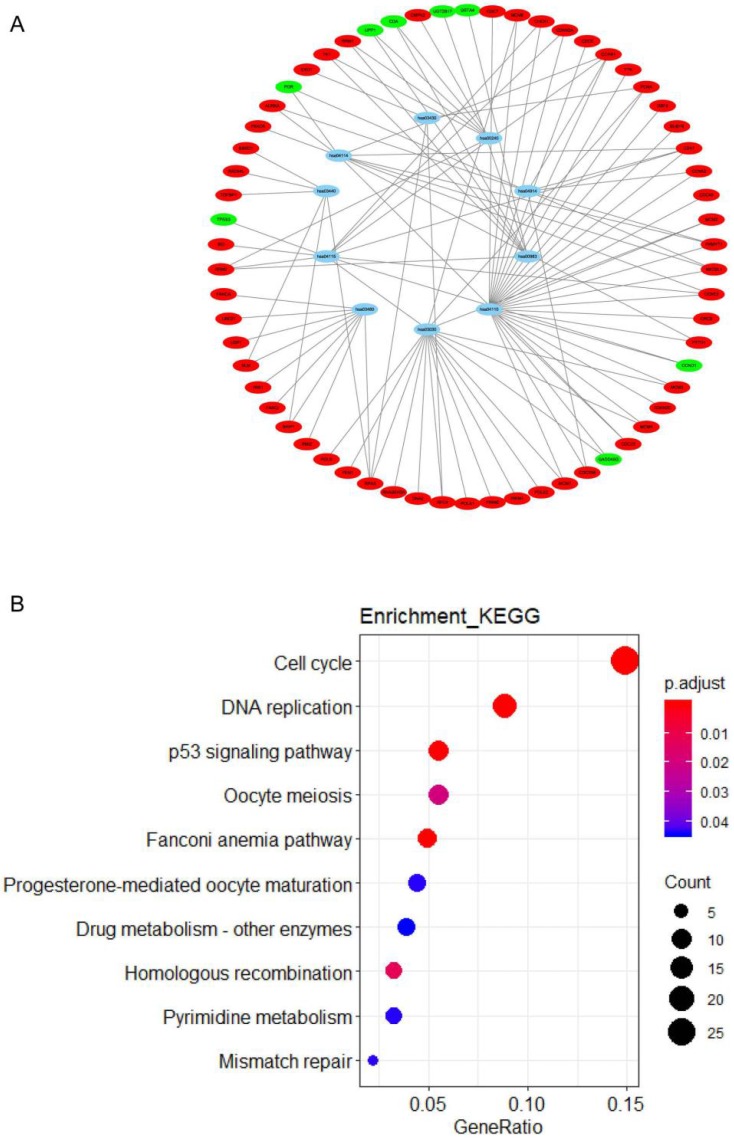
Significantly signaling pathway analysis of differentially expressed genes (DEGs) related to CIN performing with KEGG pathway website and software R. (A) The network of pathways and genes, blue represents pathways, green is the down-regulated gene, red is the up-regulated gene. (B) Pathway enrichment analysis based on differentially expressed genes (DEGs). GeneRatio = count/setsize.

All DEGs were uploaded to the online website DAVID to discern GO classfication. The terms for each GO category were shown in Fig 2 and Fig S2. The most DEGs were enriched in chromosomal region in CC (Fig. 2A), organelle fission in BP (Fig. 2B) and ATPase activity in MF (Fig. 2C). The results of pathway enrichment analysis were shown in Fig. 3.

**Figure 4:**
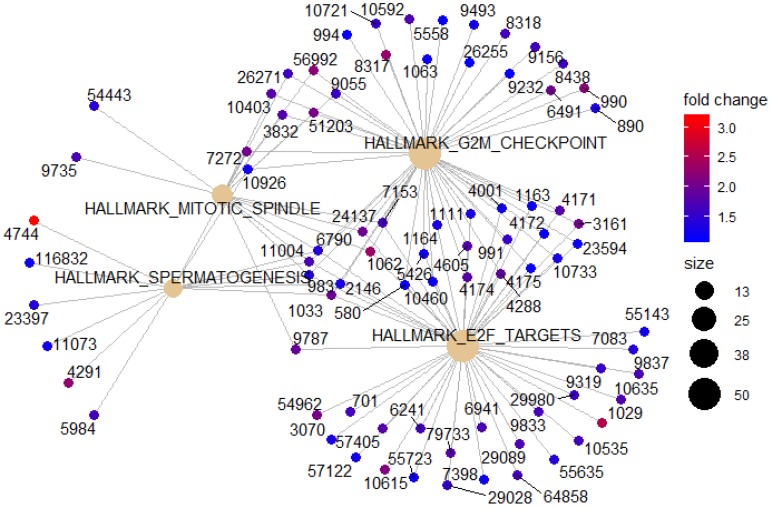
GESA Constructs function set and genes network. Yellow represents functional sets, the number on the outer edge of the network represents entrezID.

**Figure 5:**
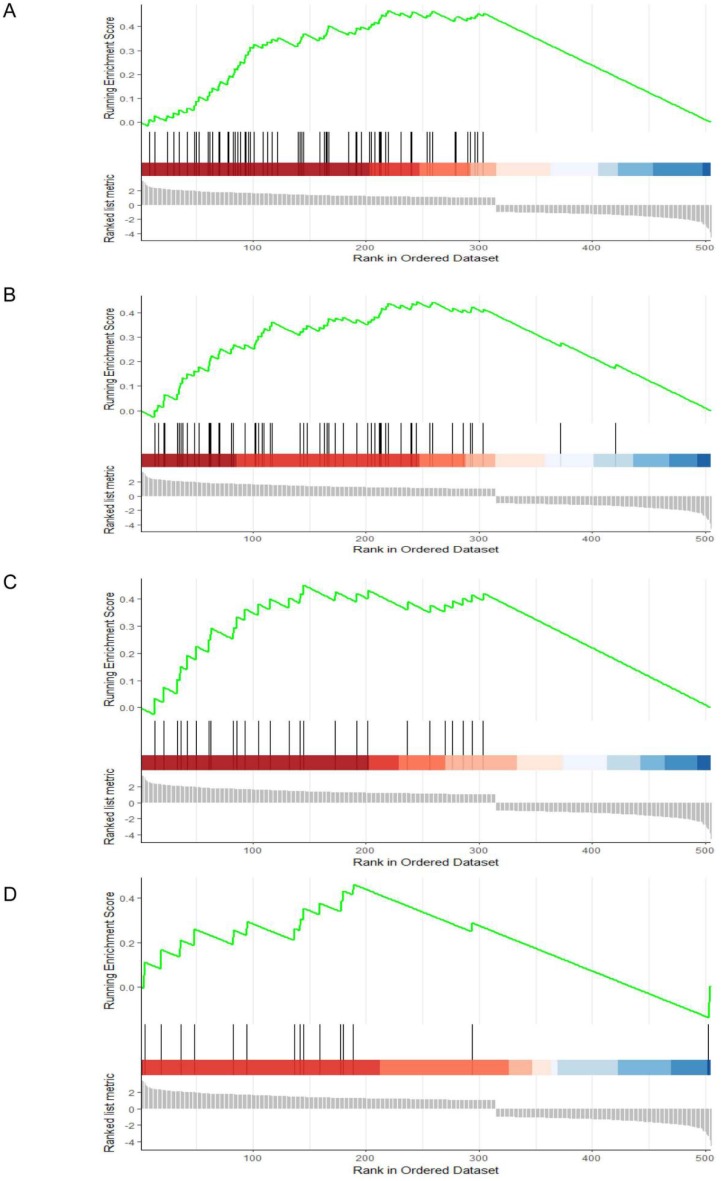
Gene set enrichment analysis (GSEA). (A) E2F-Targets (B) G2M-Checkpoint (C) Mitotic-Spindle (D) Spermatogenesis

To identify potential function of the hub genes, GSEA was conducted respectively to search “All gene sets” enriched in the samples with the gene highly expressed. The DEGs are mainly enriched in 4 functional sets: E2F-Targets, G2M-Checkpoint, Mitotic-Spindle and Spermatogenesis (Fig. 4 and 5).

### Co-expression network construction and key modules identification

**Figure 6:**
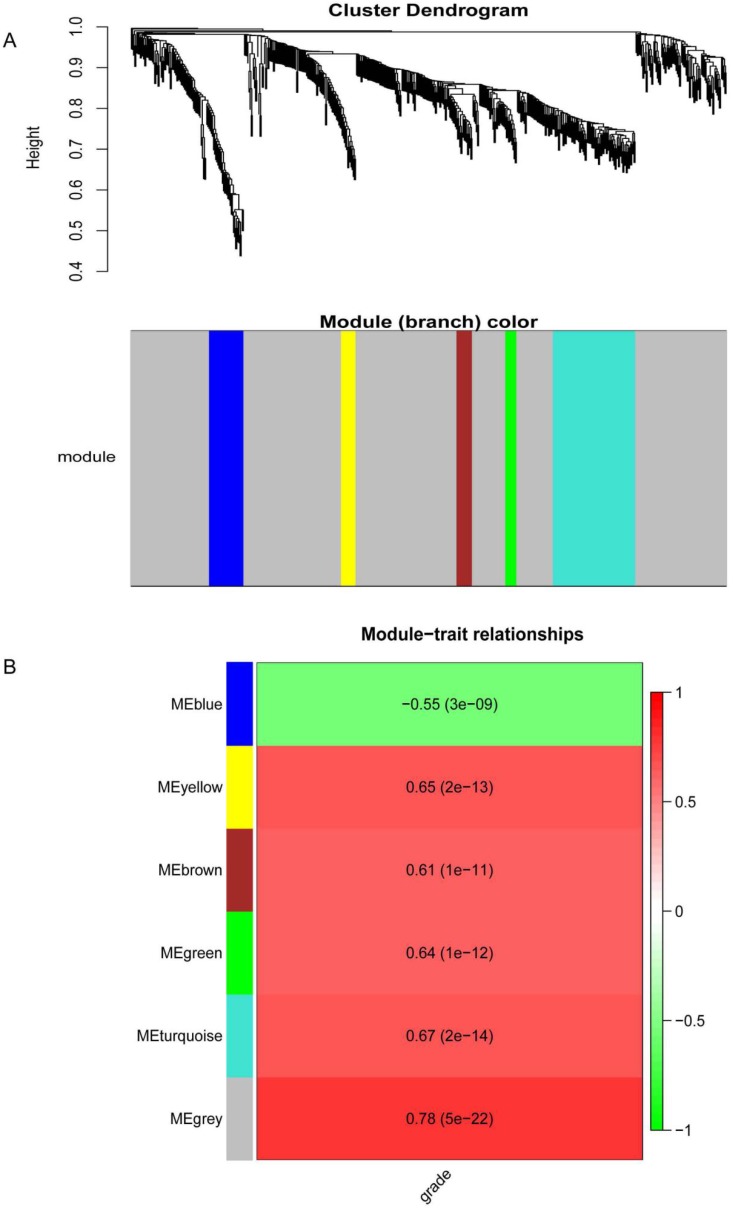
Results of the co-expression network.(A) Dendrogram of the differentially expressed genes (DEGs) of GEO datasets clustered. (B) The correlation between the module eigengenes and the CIN grade.

**Figure 7:**
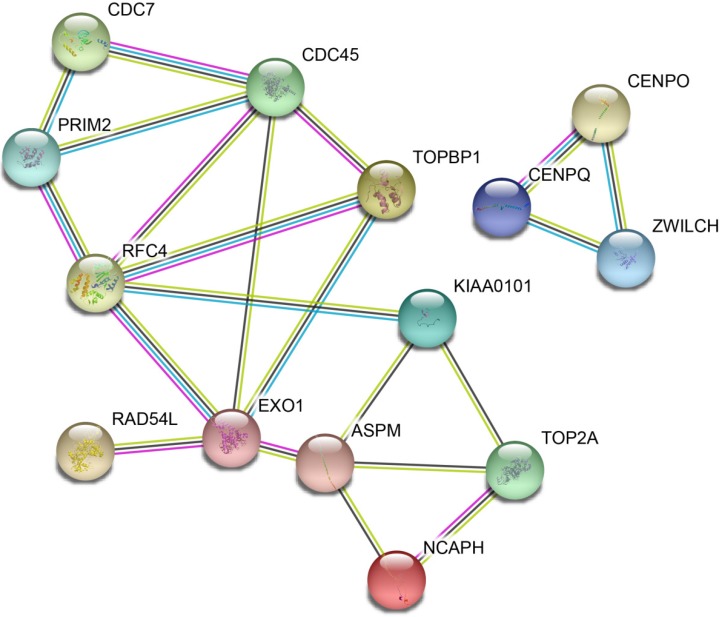
Protein-protein interaction (PPI) network of differentially expressed genes (DEGs)

**Figure 8:**
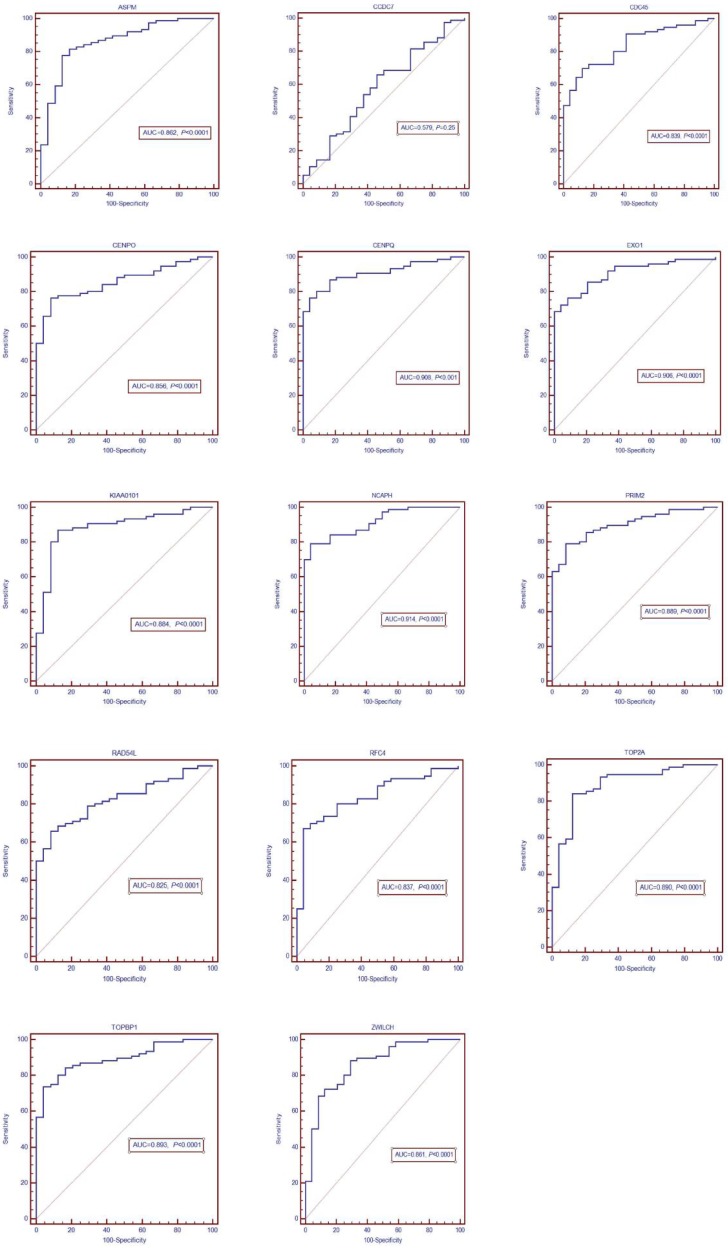
ROC diagnosis analysis of the differentially expressed genes (DEGs) for CIN

The DEGs with similar expression patterns were grouped into modules via the average linkage hierarchical clustering, calculated by “WGCNA” package. A total of 6 modules were identified (Fig. 6A). Subsequently, we calculated the correlation between gene module and CIN grade. Grey module has the highest correlation (Cor=0.78, *P*=5e-22; Fig. 6B). Therefore, 31 genes with the high connectivity in grey module were taken as candidate hub genes for CIN high grade risk in the module (weighted correlation coefficients >0.80, Tab S2). The analysis of protein interaction network suggested that 14 of these genes might interact more closely in CIN classification (Fig. 7 and Tab S3). Diagnostic analysis results showed that in addition to CCDC7, the expression levels of the remaining 13 genes have a high diagnostic value (AUC>0.8 and *P*<0.05; Fig. 8).

## Conclusion

DEGs in CIN samples can be used to diagnose the progressing disease before it leads to cancer. A combinatorial approach utilizing gene expression profile, PPI network, hubs, modules and motifs was employed to identify potential prognostic markers capable of distinguishing progressing cervical disease. A total of 537 DEGs (331 up-regulated genes and 206 down-regulated genes) were identified in CIN samples by gene expression profiling. These genes also deregulated a number of biological pathways including: Cell cycle, DNA replication, Fanconi anemia pathway, p53 signaling pathway, Homologous recombination, Oocyte meiosis, Mismatch repair, Pyrimidine metabolism, Progesterone-mediated oocyte maturation and Drug metabolism - other enzymes. In addition, 4 functional gene sets were enriched: E2F-Targets, G2M-Checkpoint, Mitotic-Spindle and Spermatogenesis. 31 DEGs out of 537 were found as candidate hub genes for CIN high grade risk. Among them, 13 genes might interact more closely in CIN classification and have a high diagnostic value.

The most DEGs were enriched in chromosomal region in CC, organelle fission in BP and ATPase activity in MF. Chromosomal instability is a crucial sign of malignancy. Kudela E et al.^16^ focused on chromosomal changes in the process of cervical carcinogenesis and CIN. This study indicated the amplification of chromosomal regions increases with the degree of dysplasia toward the invasive disease. Increasing in the amplification of 3q26 is noticeable already at CIN 2 + lesions, and 5p15 amplification is shifted up toward CIN 3. At present, organelle fission focuses on mitochondrial fission. Mitochondria are highly dynamic organelles, and mitochondrial fission is a crucial step of apoptosis^17^. Mitochondrial fragmentation is involved in the apoptotic process of cervical cancer^17^. However, whether this is related to CIN has not yet been clarified. As a condition in which cells change their chromosomal content at a high rate, chromosomal instability is a consistent feature of the majority of solid tumours^18^, and chromosomal instability plays an important role in cervical disease, and is significantly associated with patient outcome. KEGG results showed that most DEGs enrichment pathways were related to cell cycle. Ki67 is a marker of cell proliferation, and the increased expression of Ki67 is correlated with higher cervical CIN grade and is a highly sensitive biomarker for differentiating between CIN1 and CIN2/3^19^,^20^. In addition, high-risk HPV E7 oncoproteins bind and inactivate pRb, leading to abnormal cell proliferation^21^.

Previous studies have focused on different types of solid tumors (cervical cancer), such as genetic instability at gene locus 1p36, which may be a feature of cervical cancer^22^; decreased expression of cytokeratin 7 may lead to poor prognosis of cervical cancer^23^; HPV infection is an important potential biomarker of cervical cancer^24^; neutrophil ratio and white matter cell count can be used as a prognostic factor for recurrence of cervical cancer^25^. However, the continuous process from inflammation to CIN to invasive cancer is often overlooked. Since CIN is the most important precancerous lesion of cervical cancer, we focus more on the progress from normal cervical epithelial tissue to CIN, which is closely related to the occurrence and progression of cancer. Our results show that TOP2A and RFC4 play an important role in this process. TOP2A is regarded as a biomarker for the improved diagnosis of CIN^26^. Recent study has shown that TOP2A protein is expressed in cells with aberrant S-phases and including HPV-transformed cells in association with elevated expression of the HPV E6/E7 proteins^27^.

It is worth noting that many studies have shown that TOP2A expression level is significantly correlated with CIN grade^26^,^28^. In addition, RFC4 accelerated G1 to S phase progression, and promoted the proliferation of cervical cancer cells and the growth of cervical cancer^29^. However, our study screened 13 DEGs related to CIN grade. At present, there is not enough evidence to support the association with CIN grade except TOP2A and RFC4. The research of gene bioinformatics provides a possible molecular targeting mechanism for the treatment of progressive cervical diseases. Therefore, subsequent studies will focus on validating these DEGs.

The limitation of this study is that the data used in this study are from public databases, so the quality cannot be evaluated. In addition, we did not further study the differential expression of CIN to cervical cancer.

To sum up, this study used bioinformation-based methods to reveal DEGs related to CIN. This study is a gene analysis with a large sample size that integrates microarray data from GEO databases. Then the functional and pathway enrichment analysis of DEGs was carried out. In addition, the WGCNA method was used to analyze the clinical data graed related to CIN. Therefore, this research provided a new sight into the understanding of molecular functions for CIN. However, further experiments are required to confirm and validate these predicted results.

## Supplementary Material

Supplementary figures and tables.
