# Supplementary material for: Bioinformatics analysis and identification of potential genes related to pathogenesis of cervical intraepithelial neoplasia
Source: J Cancer. 2020 Feb 3;11(8):2150–7. doi: 10.7150/jca.38211 (PMC7052918; doi:10.7150/jca.38211)
Supplement: Supplementary file 1 — Supplementary figures and tables. [file jcav11p2150s1.pdf]

Figure S1 Volcano plot of differentially expressed genes (DEGs). Red, green and black denote up-regulated, down-regulated and non-significantly expressed genes, respectively.

Figure S2 Gene and functional network constructed by GO enrichment analysis.

Figure S1

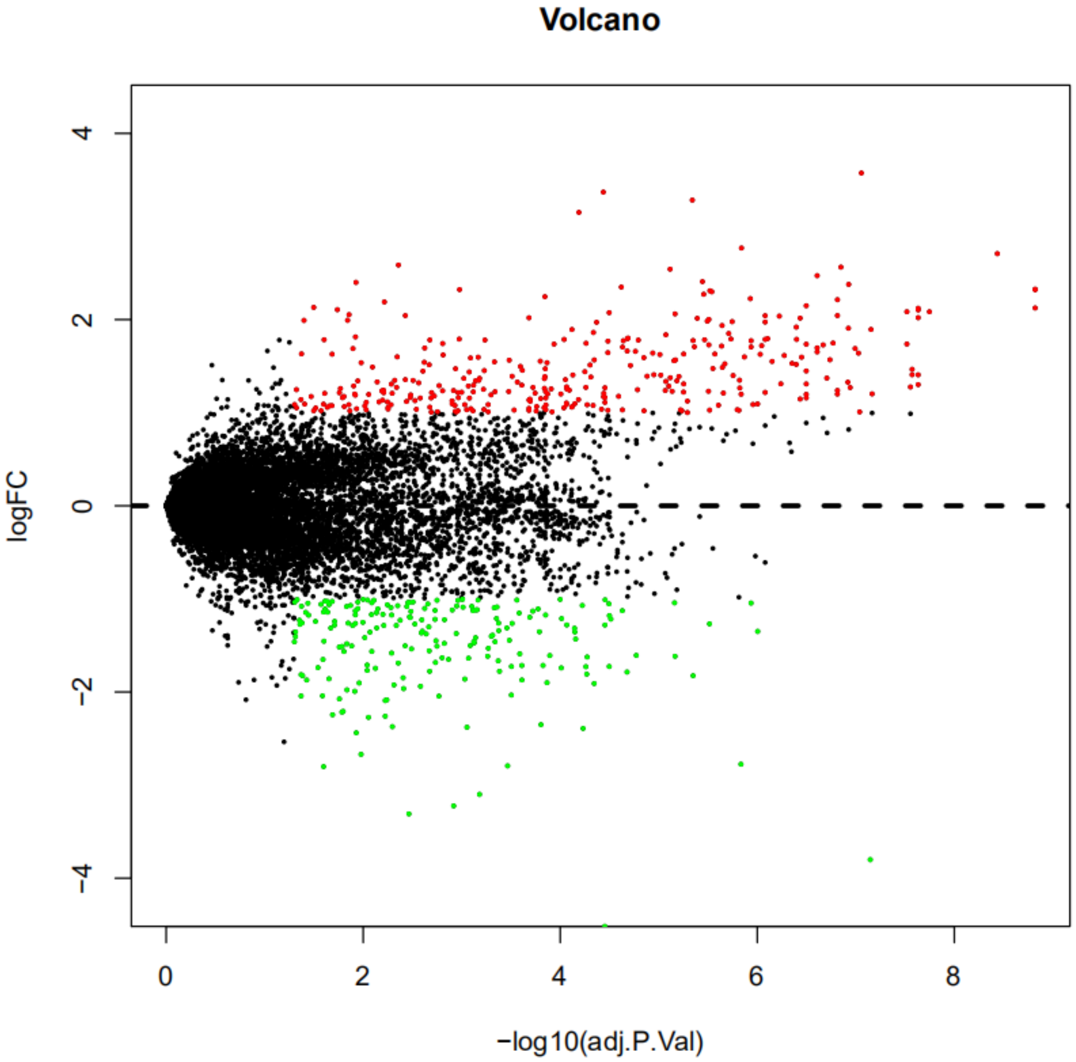

Figure S2

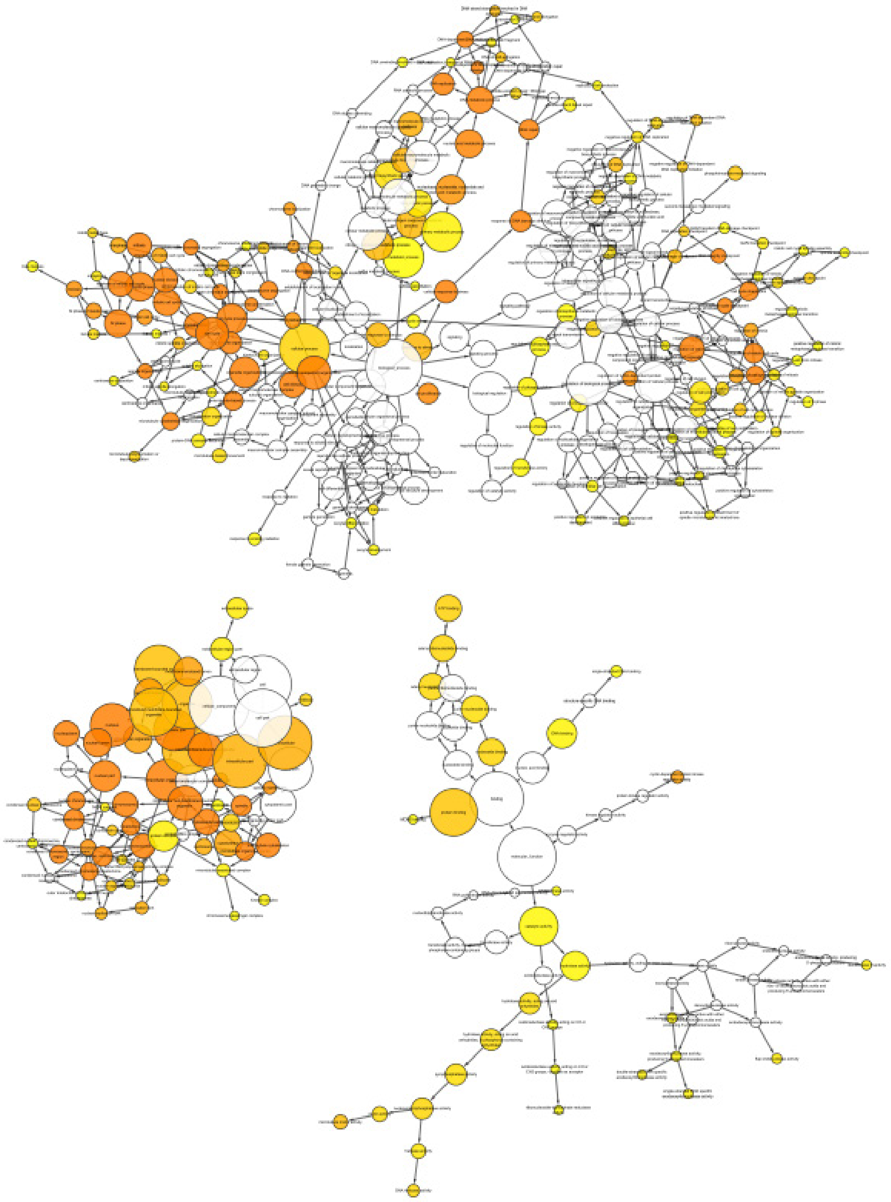

Table S1 Clinical evaluation of cervical specimens

	Normal (n=24)	CIN1 (n=14)	CIN2 (n=22)	CIN3 (n=40)
**Median age**	28.5	27	24	30.5
**HPV genotype**				
HPV 16	16 (16.2)	12 (12.1)	20 (20.2)	32 (32.3)
HPV 18	1 (25.0)	0	0	2 (50.0)
other high-risk HPV	5 (25.0)	2 (10.0)	2 (10.0)	6 (25.0)
other HPV	2 (40.0)	0	0	1 (20.0)
**Study cytology**				
HSIL+	16 (14.3)	13 (11.6)	22 (19.6)	37 (32.1)
LSIL	3 (50.0)	1 (16.7)	0	2 (33.3)
ASC-US	2 (40.0)	0	0	2 (40.0)
Normal	3 (100.0)	0	0	0
Missing	0	0	0	0
**Colposcopy impression**				
Cancer	0	0	1 (3.2)	8 (25.8)
High grade	7 (29.2)	0	7 (29.2)	9 (37.5)
Low grade	13 (23.6)	11 (20.0)	14 (25.5)	15 (25.5)
Metaplasia & Equivocal	3 (25.0)	2 (16.7)	0	6 (50.0)
Normal	1 (100.0)	0	0	0
Missing	0	1 (20.0)	0	3 (60.0)

HSIL = high grade squamous intraepithelial lesion; LSIL = low grade squamous intraepithelial lesion; ASC-US = atypical squamous cells of undetermined significance

Roemeling C A V , Radisky D C , Marlow L A , et al. Neuronal pentraxin 2 supports clear cell renal cell carcinoma by activating the AMPA-selective glutamate receptor-4[J]. Cancer Research, 2014, 74(17):4796-810.

Table S2 31 genes with the high connectivity in grey module were taken as candidate hub genes for CIN high grade risk

Gene	colorNEW	kMEblue	kMEbrown	kMEgreen	kMEgrey	kMEturquoise	kMEyellow
SYCP2	grey	-0.31933	0.813579	0.748354	0.902993	0.792075	0.674302
TCAM1P	grey	-0.56294	0.687622	0.70513	0.890289	0.765379	0.66542
DCLRE1B	grey	-0.57014	0.753084	0.756259	0.87688	0.789789	0.769592
CDC7	grey	-0.26862	0.860968	0.809275	0.872573	0.84401	0.704296
TOPBP1	grey	-0.30903	0.832814	0.742776	0.863863	0.871869	0.642696
ACTL6A	grey	-0.60488	0.653026	0.693032	0.86191	0.78691	0.641038
CKLF	grey	-0.48131	0.737526	0.716072	0.86049	0.822085	0.667799
C18orf54	grey	-0.5212	0.699997	0.663395	0.859988	0.783101	0.671595
CENPQ	grey	-0.62882	0.688158	0.671624	0.858939	0.817186	0.651964
ATAD2	grey	-0.23736	0.772421	0.788452	0.8506	0.79	0.547155
PRIM2	grey	-0.34016	0.786596	0.746414	0.848956	0.796391	0.641971
RAD54L	grey	-0.30243	0.886984	0.84222	0.842679	0.750092	0.825727
EXO1	grey	-0.63054	0.740341	0.723948	0.840726	0.760152	0.790412
KIAA0101	grey	-0.51815	0.739371	0.812399	0.83823	0.820286	0.697761
HENMT1	grey	-0.14286	0.777	0.733399	0.837402	0.73288	0.58583
TOP2A	grey	-0.47398	0.750826	0.848469	0.834757	0.841481	0.689668
NCAPH	grey	-0.40566	0.811809	0.873767	0.826243	0.818959	0.701439
ZWILCH	grey	-0.31397	0.783499	0.825587	0.825152	0.823406	0.622159
LINC00925	grey	-0.42662	0.649481	0.661172	0.823625	0.630425	0.627539
CENPO	grey	-0.39311	0.832394	0.781135	0.820124	0.715549	0.843433
ZFR2	grey	-0.38531	0.633341	0.665261	0.818176	0.741588	0.468172
RIBC2	grey	-0.71674	0.551642	0.599487	0.816547	0.654397	0.6715
SYNGR3	grey	-0.53921	0.535436	0.609433	0.814336	0.677193	0.509219
ASPM	grey	-0.46706	0.67391	0.728141	0.814137	0.717365	0.671502

LOC375196	grey	-0.2865	0.664535	0.677685	0.814128	0.662071	0.560577
RFC4	grey	-0.09753	0.868167	0.855795	0.813682	0.8086	0.610759
DHFR	grey	-0.18802	0.815199	0.854834	0.810412	0.830256	0.555871
CDKN2A	grey	-0.32164	0.646347	0.717055	0.806906	0.749467	0.439761
MLF1	grey	-0.25365	0.691977	0.622876	0.804038	0.674678	0.53789
CDC45	grey	-0.21746	0.869529	0.888799	0.803326	0.789017	0.706893

Table S3 Protein to Protein Interaction with high interaction score

node 1	node 2	neighborhood_on_chromosome	gene_fusion	phylogenetic_cooccurrence	homology	coexpression	experimentally_determined_interaction	database_annotated	automated_textmining	combined_score
CENPO	CENPQ	0	0	0	0	0.107	0.917	0.9	0.903	0.999
CDC45	CDC7	0	0	0	0	0.431	0.264	0.9	0.919	0.996
PRIM2	CDC45	0	0	0	0	0.863	0	0.9	0.593	0.993
PRIM2	RFC4	0	0	0	0	0.874	0.157	0.9	0.442	0.993
RFC4	EXO1	0	0	0	0	0.74	0.186	0.9	0.503	0.988
RFC4	TOPBP1	0	0	0	0	0.589	0.18	0.9	0.606	0.984
CDC45	TOPBP1	0	0	0	0	0.26	0.829	0	0.86	0.98
EXO1	TOPBP1	0	0	0	0	0.271	0	0.9	0.694	0.975
RFC4	KIAA0101	0	0	0	0	0.757	0	0.9	0.09	0.975
TOP2A	ASPM	0	0	0	0	0.967	0	0	0.234	0.973
TOP2A	KIAA0101	0	0	0	0	0.949	0	0	0.263	0.961
TOP2A	NCAPH	0	0	0	0	0.775	0.572	0	0.569	0.954
CENPO	ZWILCH	0	0	0	0	0.098	0	0.9	0.512	0.952
CENPQ	ZWILCH	0	0	0	0	0.194	0	0.9	0.441	0.951
CDC45	EXO1	0	0	0	0	0.902	0	0	0.527	0.951
CDC45	RFC4	0	0	0	0	0.852	0.064	0	0.658	0.948
PRIM2	CDC7	0	0	0	0	0.213	0	0.9	0.273	0.937
RAD54L	EXO1	0	0	0	0	0.75	0.147	0	0.699	0.93
ASPM	EXO1	0	0	0	0	0.918	0.104	0	0.089	0.927

ASPM	NCAPH	0	0	0	0	0.903	0	0	0.15	0.914
ASPM	KIAA0101	0	0	0	0	0.903	0	0	0.068	0.906
